# The evolving Lebanese drug crisis: Trends in drug availability and affordability for common outpatient diseases from 2019 to 2023

**DOI:** 10.1371/journal.pgph.0002538

**Published:** 2023-11-01

**Authors:** Rachel Jaber Chehayeb, Shashwat Kala, Huwayda Abou Ghannam, Ghassan Hasan, Joe Salloum

**Affiliations:** 1 Yale School of Medicine, New Haven, CT, United States of America; 2 Independent Researcher, Community Pharmacist, Huwayda’s Pharmacy, Aley, Lebanon; 3 Independent Researcher, Community Pharmacist, Al Ikha Pharmacy, Kabr Shmoun, Lebanon; 4 Beirut Arab University, Beirut, Lebanon; 5 Lebanese Order of Pharmacists, Beirut, Lebanon; Institute for Epidemiology, Biostatistics and Prevention, University of Zurich, SWITZERLAND

## Abstract

Since 2019, Lebanon has been suffering from an enduring economic crisis, that in conjunction with the COVID-19 Pandemic and the Beirut Port Explosion, has had catastrophic consequences on many facets of the Lebanese healthcare system. However, few studies have operationalized the impact of the crisis on drug availability and affordability. This is particularly relevant given that Lebanon imports approximately 95% of pharmaceutical products. Toward this end, we evaluated trends in outpatient drug availability and affordability in the context of monthly mean income at three time points throughout the evolving economic crisis (pre-crisis-August 2019, early crisis- August 2021, most recent-April 2023). Drugs used to treat the most common causes of mortality in Lebanon were selected from the Lebanese Ministry of Public Health (MOPH)’s List of Essential Medications. Drug pricing was obtained from the Lebanese MOPH National Drug Database. We found large increases in drug prices, as a percentage of mean monthly income, after subsidies on chronic disease medications were removed. Diabetes and COPD drugs were the least affordable in 2023, amounting to 21.03% and 15.43% of mean monthly income, respectively. We also highlight great shortages in drugs across classes, particularly in mood-stabilizing psychiatric drugs and basal and bolus insulin. Our findings highlight the growing financial burden of chronic disease treatment in Lebanon and the importance of implementing both short- and long-term solutions to mitigate the impact of the crisis on public health.

## Introduction

Since the start of the Lebanese liquidity crisis in October 2019, Lebanon, previously a high-middle income country, has suffered from a progressively worsening economic crisis which has pushed greater than 80% of the population into poverty. According to the World Bank, this crisis ranks among the top three most severe economic crises since the mid-nineteenth century [1–3]. The crisis has been further exacerbated by the COVID-19 pandemic, the 2020 Beirut Port explosion, and political instability. These factors, among others, have resulted in a greater than 95% devaluation of the Lebanese Pound (LBP) [4]. Prior to the crisis, from 1997 to August 2019, the LBP was pegged to the US dollar with a conversion of 1USD = 1,507.5 LL. In the past three years, however, the LBP has undergone around a 63-fold devaluation, with a current black-market exchange rate of 1USD to 94,500 LBP (May 9^th^, 2023).

The economic crisis has impacted every economic sector and all aspects of life in Lebanon. For example, in an August 2022 field survey of 931 Lebanese households across governorates, 29% of respondents reported losing their job since the onset of the crisis, 85% purchased fewer food staples, 75% reduced automobile use due to cost of gasoline, 86% turned off indoor heating despite being cold, and 33% skipped meals [5].The survey also highlights the crisis’s impact on the healthcare sector, with 63% of respondents reporting skipping or postponing doctor visits when ill, 59% reporting shifting from private to public healthcare, and 31% reporting discontinuing a prescription medication [5].

The crisis has impacted the functioning of hospitals in a variety of domains: shortages in foreign currency limiting the ability to import medical products, brain drain, staffing shortages, bankruptcy, and inability of patients to pay for medical care [6]. Many hospitals stopped performing non-life-threatening surgeries in the face of anesthetic shortages [7]. These challenges have led to marked psychological disturbances on healthcare workers as indicated by increased scores on the Generalized Anxiety Disorder 7 (GAD-7) and Patient Health Questionnaire (PHQ-9) [8].

The economic crisis has also greatly impacted access to pharmaceutical products. The severe inflation has resulted in soaring drug prices. In addition, given that Lebanon imports 95% of all pharmaceutical products, the devaluation of the currency has brought forth devastating shortages in medications due to a limited ability to import drugs and raw materials from foreign suppliers [9]. Since 2021, the country has suffered shortages across drug classes ranging from life-saving chemotherapy to over-the-counter pain killers [10].

To mitigate the difficulty faced by drug suppliers in exchanging LBPs to USD, the Central Bank initiated a subsidy system to increase drug availability in November 2019. The Central Bank subsidized 85% of the USD value needed to buy medicines, requiring drug importers to fund the remaining 15% USD value at the LBP-to-USD black-market exchange rate[4, 10, 11]. The effectiveness of this system was limited by the smuggling of subsidized drugs out of the country to sell for profit, the stockpiling of medications, and delays in subsidy request processing [11]. Moreover, given the worsening liquidity crisis, the subsidy program proved unsustainable. On November 9^th^, 2021, the Ministry of Public Health (MOPH) discontinued subsidies on all medications except for oncology drugs and certain drugs for chronic disease management [11]. Moreover, the mounting debt that Lebanese drug importers owed international pharmaceutical companies further limited their ability to import drugs regardless of subsidy status [11]. Consequently, drug prices started to increase significantly [11].

The crisis, continuous rapid devaluation of the LBP, and evolving MOPH subsidies have resulted in a rapid change in the landscape of pharmaceutical drugs in Lebanon. However, few studies have quantified the changes in drug affordability and availability over the past three years. Our paper aims to fill this gap and evaluate trends in outpatient drug affordability and availability in the context of monthly mean income at three time points throughout the evolving economic crisis (pre-crisis-August 2019, early crisis- August 2021, most recent-April 2023).

## Methods

### Study design

To evaluate trends in drug availability and affordability immediately prior to and during the evolving Lebanese drug crisis, three timepoints were selected for analysis. The first, August 2^nd^ 2019, corresponds to a date immediately prior to the onset of the economic crisis; the second, August 17^th^ 2021, represents the date when subsidy status was first listed on the Lebanese National Drug Database list; and the third, April 3^rd^ 2023, was the most recent date with available data at the time of analysis [12].

### Drug selection

The most common causes of mortality in Lebanon from 2009 to 2019 were obtained from the Institute for Health Metrics and Evaluation [13]. From this list, we selected the most common outpatient diseases and subsequently generated the following five drug classes used to treat these conditions: antibiotics, anticoagulant & antithrombotics, cardiovascular, diabetes, and COPD. Though not a leading cause of mortality in Lebanon, we chose to also include psychiatric medications as a separate drug class due to the increasing rates of mental health disorders associated with the economic crisis, the 2020 Beirut explosion, and the COVID-19 pandemic [14, 15]. A final list of medications for analysis was selected in a two-step process: 1) medications included in the 2018 Lebanese Ministry of Public Health list of essential medications that fit within the drug classes listed above; 2) medications not included in the 2018 list that were deemed relevant to treat the conditions listed above [16]. Table 1 outlines these six drug classes and the medications within each class.

**Table 1:** Drug included in each class.

Drug Class	Drugs Included
Antibiotics	amoxicillin, amoxicillin + clavulanic acid, cefalexin, cefixime, azithromycin, ciprofloxacin, doxycycline, ceftriaxone, metronidazole, nitrofurantoin, norfloxacin, sulfamethoxazole + trimethoprim, erythromycin, clindamycin
AC/ antithrombotic	acenocoumarol, enoxaparin, phytomenadione, desmopressin, rivaroxaban*, dabigatran*, acetylsalicylic acid, clopidogrel, calcium dobesilate, apixaban*,
Cardiovascular Drugs	bisoprolol, atenolol, glyceryl trinitrate, isosorbide dinitrate, molsidomine, propranolol, verapamil, digoxin, amiodarone, amlodipine, diltiazem, ramipril, hydrochlorothiazide, valsartan, losartan, methyldopa, captopril, enalapril, furosemide, spironolactone, fenofibrate, atorvastatin, simvastatin, rosuvastatin, gemfibrozil, hydrochlorothiazide, amiloride hcl, indapamide
Diabetes	gliclazide, glimepiride, intermediate-acting insulin 70/30, long-acting insulin n, rapid-acting insulin r, metformin, vidagliptin*, sitagliptin*, dapagliflozin*, canagliflozin*,empagliflozin*, insulin lispro*, insulin glulisine*, insulin degludec*, insulin detemir*, insulin glargine*,
Psychiatry	chlorpromazine, haloperidol, risperidone, zuclopenthixol decanoate, clozapine, amitriptyline, fluoxetine, sertraline, imipramin, carbamazepine, lithium carbonate, valproic acid, clonazepam, diazepam, clomipramine
COPD Drugs	beclometasone, budesonide, budesonide + formoterol, ipratropium bromide, montelukast, salbutamol, aminophylline, dextromethorphan, tiotropium bromide*, fluticasone*, fluticasone + formoterol *, fluticasone furoate + vilanterol*,

* = drugs not included in the 2018 Lebanese Ministry of Publich Health’s List of Essential Medications but included in the 2018 World Health Organization’s List of Essential Medications that were deemed important to common chronic outpatient conditions

#### Sources of drug pricing and subsidy status

Information regarding the price and subsidy status for doses of each medication at each timepoint was obtained from the Lebanese National Drug Database [12], which the MOPH updates regularly. For antibiotics, listed costs are that of the most prescribed dosage for a single course treatment. For chronic disease medications, costs correspond to a one-month supply, except when limited by medication manufacturing and packaging. For example, lithium carbonate is only present in 100 tablet boxes (S1 Table).

#### Source of drug availability data

Despite several attempts, we were unable to find any public data on drug availability. No such data is available on the Ministry of Public Health’s nor the Order of Pharmacists’ websites. We contacted officials at the Lebanese Order of Pharmacists and at the Lebanese Syndicate of Drug Importers who confirmed that national data around drug availability over the past three years exists. Hence, we resorted to the expertise of local community pharmacists.

Drug availability was independently compiled via two community pharmacists (authors HAG and GH) based on their experience communicating with importers and their pharmacy records indicating stock shortages at different time points over the past three years. Specifically**, they** have been in daily communication with national drug importers and manufactures to determine drug availability throughout the evolving economic crisis. Their compiled data was then corroborated by the President of the Lebanese Order of Pharmacists, author JS.

### Statistical analysis

We performed descriptive statistics and calculated mean relative price increases between 2019, 2021, and 2023. Additionally, we compared the mean cost of medications within each drug class to the monthly mean income (MMI).

Given the absence of governmental data on mean individual and household incomes and the daily devaluation of the LBP, calculating monthly incomes required extrapolation from existing data [17]. To achieve this, we used the International Labor Organization’s (ILO) report on mean income in 2018–2019 as our pre-crisis income (August 2019). However, there was no listed income for August 2021 and April 2023. To estimate these values, we used the mean income in LBP from January 2022, the most recently available data within the same ILO report. From there, we used the LBP to USD exchange rates at each time point to determine mean monthly income at August 2021 and April 2023 assuming a fixed salary in dollar value.

## Results

Drug availability and pricing from 2019 to 2023 across six drug classes indicated for the treatment of common outpatient diseases were evaluated.

Overview of Drugs Prices from 2019 to 2023

**Fig 1:**
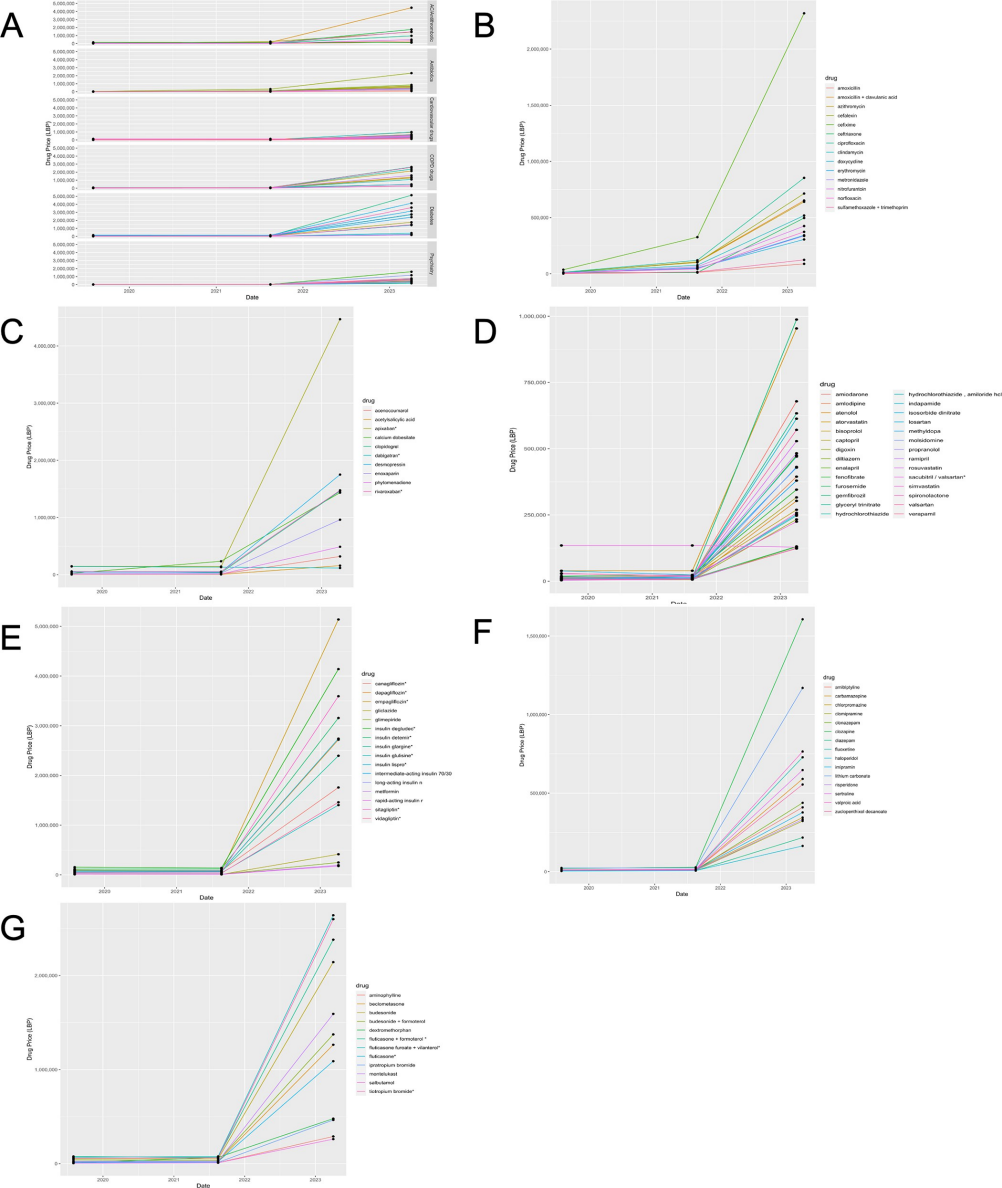
Graph showing Drug Prices in LBP as a Function of Time across Three Time Points (Pre-Crisis-August 2019, Early Crisis- August 2019, Most Recently-April 2023): **A**) All Drugs Classes, **B)** Diabetes, **C)** Anticoagulants/ Antithrombotics, **D)** Cardiovascular Drugs, **E)** Diabetes Drugs, **F)** Psychiatric Drugs, **G)** COPD Drugs.

Fig 1A depicts changes in drug cost in LBP across these time points for all listed drugs within each of the six categories. A general trend emerges from this figure: a minimal increase in mean drug price from August 2019 to August 2021 and a much more drastic increase in mean drug price from August 2021 to April 2023.

### Antibiotics

Fig 1B shows that within the outpatient antibiotics class, cefixime was the most expensive in April 2023, at 2,318,676 LBP (24.5USD) for a standard course, followed by ciprofloxacin at 853,483 LBP (9.0 USD). To put these numbers in content, mean monthly income amounts to 90 USD.

### Anticoagulants and antithrombotics

Fig 1C addresses anticoagulants and antithrombotics. Notably, within this class, apixaban, one of the most widely used direct oral anticoagulants, emerges as particularly unaffordable, at an April 2023 price of 4,468,003 LBP (47.3USD) per month. Acetylsalicylic acid (aspirin), however, remains more affordable at 158,754 LBP (1.7USD). One seeming exception to the general trend is dabigatran, that has a listed price in April 2023 of 117,078 (1.3), down from 130,997 LBP in August 2021. However, this drug is no longer available in Lebanon as of 2021 (S1 Table); therefore, the listed price has likely not been updated on the MOPH website.

### Cardiovascular drugs

Fig 1D depicts trends in cardiovascular drugs. The two most expensive drugs in April 2023 in this class are gemfibrozil at 987,476 LBP (10.45USD) and atorvastatin at 953,770 LBP (10.1USD). For one cardiovascular drug, sacubitril/valsartan, the price appears to plateau across timepoints. However, as seen in S1 Table, it has been in shortage since 2021 and remains completely unavailable as of April 2023.

### Diabetes drugs

Fig 1E shows remarkable increases in diabetes drugs across timepoints. Within insulins, insulin degludec (5 pens of 300 units) costing 4,139,738 LBP (43.8 USD) was the most expensive drug in April 2023, followed by insulin detemir (5 pens of 300 units) costing 3,156,519 LBP (33.4 USD). In general, the price of both long and short acting insulins markedly increased after subsidies were removed. Dipeptidyl peptidase-4 inhibitors (DPP-4) and sodium glucose transporter 2 (SGLT2) inhibitors are also particularly unaffordable with a month supply of sitagliptin costing 3,594,531 LBP (38.0 USD) and empagliflozin costing 5,138,373 LBP (54.4 USD).

### Psychiatry drugs

As depicted in Fig 1F, the most expensive psychiatric drug in April 2023 was clozapine at 1,606,470 LBP (17.0 USD) followed by lithium carbonate at 1,169,533 LBP (12.4 USD). Haloperidol has remained the cheapest at 163,955 LBP (1.7 USD).

### COPD drugs

Finally, within the COPD drug class (Fig 1), the two most expensive drug in April 2023 were fluticasone furoate + vilanterol costing 2,640,307 LBP (28.0 USD) and tiotropium bromide at 2,598,680 LBP (27.5 USD).

### Trends in drug prices

**Fig 2:**
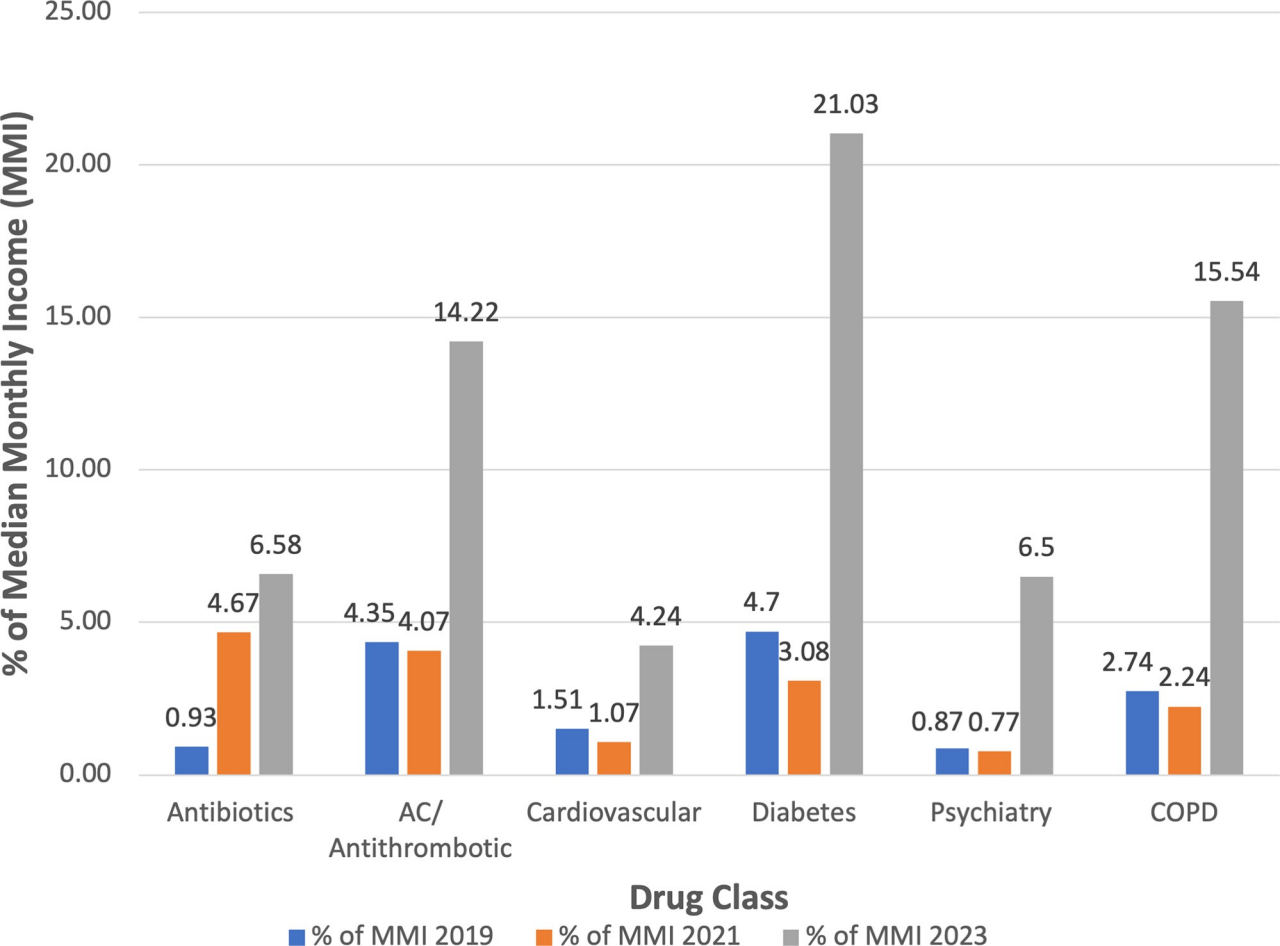
Bar graph illustrating the percent of mean monthly income that the mean monthly drug price per class represents across the three time points (pre-crisis-August 2019, early crisis- August 2019, most recently-April 2023).

Next, changes in drug price were compared to mean monthly incomes (MMI) across timepoints, as depicted in Fig 2 and Table 2. As these indicate, in 2019, psychiatry drugs (0.87% of MMI) and diabetes drugs (4.7%) were the most and least affordable, respectively. In 2021, while psychiatry drugs (0.77%) remained the most affordable and antibiotics (4.67%) became the least affordable. In 2023, cardiovascular drugs (4.24%) and diabetes drugs (21.03%) were the most and least affordable, respectively. Additionally, psychiatry drugs experienced the largest increase in percentage of MMI (7.47-fold) from 2019 to 2023 while cardiovascular drugs had the smallest increase (2.81-fold). Across all drug categories, we report large standard deviations reflecting the large price variation between different medications within each drug class (Table 2). Once again, overall trends mimic those seen in Fig 1A–1F, with minimal changes in % MMI occurring from 2019 to 2021 and more drastic changes in % MMI occurring from 2021 to 2023. In fact, all but one drug class decreased in % MMI between 2019 and 2021, with antibiotics emerging as a notable exception due to their lack of subsidization.

**Table 2:** The mean drug price by class, percent of mean monthly income (MMI) that the mean price represents across the three time points, and ratio of % of MMI in 2023 to % MMI in 2023 (pre-crisis-August 2019, early crisis- August 2019, most recent-April 2023). SD = Standard Deviation.

		August 2019			August 2021			April 2023		
Drug Class	*Mean Price*	*SD*	*%MMI*	*Mean Price*	*SD*	*%MMI*	*Mean Price*	*SD*	*%MMI*	*%MMI 2023/ %MMI 2019*
Antibiotics	11021	7999.92	0.93	80348.5	79360.02	4.67	585044.36	543650.29	6.58	7.08
AC/ Antithrombotics	51270.1	53388.26	4.35	70127.4	75385.75	4.07	1263618.4	1279778.8	14.22	3.27
Cardiovascular	17871.97	24627.55	1.51	18366.59	23548.68	1.07	376877.28	241269.77	4.24	2.81
Diabetes	55489.25	45641.40	4.7	53109.69	42062.07	3.08	1868884.38	1607485.4	21.03	4.47
Psychiatry	10307.13	5605.63	0.87	13239.27	5913.48	0.77	577735.73	381785.74	6.5	7.47
COPD	32291.08	26139.42	2.74	38622.92	24743.82	2.24	1381345	898939.14	15.54	5.67

### Changes in drug subsidization status

As illustrated in S1 Table, in August 2021, most drugs aside from antibiotics were subsidized. However, from 2021 to 2023 there was a decrease in the number of drugs receiving government subsidies. In April 2023, only a few drugs remained subsidized, and even among subsidized drugs the degree of subsidization has significantly reduced. Partially subsidized drugs include rivaroxaban (45% subsidy), canagliflozin (65%), fluticasone+formoteral (45%), fluticasone furoate + vilanterol (45%). For a handful of other drugs, including bisoprolol, verpamil, fenofibrate and metformin, raw materials were 100% subsidized in April 2023. Of note, insulin, in both long acting and short-acting forms, has been in shortage since 2021. The same applies to several mood stabilizing psychiatric drugs such as lithium carbonate and valproic acid that have been in shortage for the past 2 years.

## Discussion

To our knowledge, this economic evaluation is the first to illustrate drug availability and affordability relative to mean income by outpatient drug class since the start of the Lebanese economic crisis. As evidenced by Fig 1, drug prices increased in the setting of the devaluation of the LBP. More importantly, comparing these price increases to change in mean income illustrates the extent to which these essential outpatient drugs have become unaffordable, highlighting the dire need for intervention (Fig 2). We identified drugs for diabetes, anticoagulation, and COPD as being particularly financially burdensome, with mean monthly cost representing 15–20% of mean monthly income. If we were to instead compare drug prices relative to minimum monthly wage, which in April 2023 was approximately a quarter of mean monthly income (2,600,000 LBP), the cost of monthly insulin easily exceeds minimum wage [18]. Our findings, along with this example, stress how Lebanon’s economically disadvantaged and most vulnerable populations are disproportionally impacted by the crisis.

In addition to drug prices, our work also assessed drug availability during the evolving crisis, as shown in S1 Table. Our results indicate severe shortages across all drug classes; notable examples include all forms of insulin and mood stabilizing drugs. As mentioned in the introduction, reasons for these drug shortages include liquidity problems, shortage of foreign currency, and debt owned by local importers [11]. Of note, these shortages have existed in both subsidized and unsubsidized medications.

The relationship between drug subsidization and availability is nuanced. For subsidized drugs, lack of liquidity from the Central Bank, on which drug importers are highly dependent, resulted in great delays in drug importation. Gradually decreasing the degree of subsidization partially alleviated the dependency on the Central Bank allowing for certain drugs that were initially in shortage to become more available, albeit at a greatly increased price. Such was the case with several antihypertensives and diuretics, such as amlodipine, ramipril, furosemide and spironolactone. Overall, this relationship explains why, across drug classes, there was a multi-fold increase in drug price relative to MMI from 2021 to 2023 (Fig 2). Cardiovascular drugs, which were subsidized to the greatest extent, experienced the lowest increase in price (Fig 2). Finally, relevant to both subsidized and unsubsidized drugs, importers owe international drug companies large sums of money. As of July 2022, the Lebanese Syndicate of Drug Importers collectively owed 400 million USD to companies [11]. The mounting debt has limited the ability to purchase drugs regardless of subsidy status.

Unsurprisingly, the unaffordability and unavailability of outpatient drugs highlighted in our research has resulted in quantifiable impacts on public health. For example, rates of decompensated heart failure, recurrent myocardial infarctions secondary to dual antiplatelet therapy shortages, diabetic ketoacidosis secondary to insulin deficiency, among other preventable complications of chronic diseases have increased [1, 4, 19]. Additionally, the crisis has challenged the financial viability and sustainability of community pharmacists by severely limiting their purchasing power [20]. This challenging landscape has pushed citizens toward extreme and potentially dangerous means to secure their medications. For example, many have turned to the black market, purchasing smuggled medications that are often expired or counterfeit [10]. Unfortunately, the true long-term burden of this drug crisis on public health has yet to be determined, though it is anticipated to be severe.

Our results should be considered in the context of several limitations. Firstly, this work does not reflect all drugs indicated for the most common causes of mortality. For example, we did not include outpatient medications for breast and lung cancers (such as aromatase inhibitors), two of the leading causes of mortality in Lebanon. We excluded these medications, in part, because treatment most commonly occurs in infusion centers. However, there have been documented shortages in both oncology drugs and associated palliative and supportive drugs, which should be further explored [21]. Secondly, when calculating mean monthly income estimates for August 2021 and April 2023, we assumed a fixed dollar amount compared to January 2022 (see methods), despite ongoing devaluation of salaries in USD. Hence, our mean monthly income estimate for August 2021 is likely an underestimation, whereas that for April 2023 is likely an overestimation. Thirdly, drug pricing and availability are continuously evolving. Our findings do not demonstrate fixed costs or availability status but rather represent snapshots at three different time points. Fourthly, our study does not report on the status of inpatient antibiotics, anesthetics, surgical equipment etc. all of which have been impacted by the crisis [6]. Fifthly, as addressed in the methods section, given the lack of any accessible public information on drug availability, our data is based on the experience of three community pharmacists in their communication with importers and their pharmacy records indicating stock shortages at different time points. We recognize the bias introduced by this method, and have attempted to mitigate it through involving three co-authors ((one of whom is the President of the Lebanese Order of Pharmacists and therefore a leading expert on this topic) in verifying the accuracy of our information.

Our findings emphasize the dire need for immediate intervention.

Some short-term solutions, summarized below, have already been proposed or implemented with varying degrees of success. For example, Primary Health Care Centers (PHCCs), which are funded by the MOPH and foreign aid, currently serve as the sole source of free medical care in Lebanon. Therefore, consistent with the recommendations of Amnesty International, we believe it is imperative to increase funding for PHCCs [11]. However, PHCCs are not a replacement for fully stocked pharmacies. Toward this end, proposed solutions include only subsidizing drugs for which there is no locally produced alternative and only subsidizing generic forms when available [22]. At the same time, infrastructure for local production should be optimized by providing local pharmaceutical companies with incentives such as tax exemptions and expanding existing raw material subsidies [22]. Furthermore, the government should scale up inspection, monitoring, and auditing efforts to prevent the smuggling of subsidized drugs out of the country and the black market trade of unregulated drugs [1]. While it is not within the scope of this paper to evaluate the efficacy of these proposed solutions, we hope that our work, which highlights the disproportionate effect of increasing drug prices on Lebanon’s middle and lower classes, can serve as a catalyst to implement meaningful change.

## Supporting information

S1 Table Table listing drug doses, prices, and availability from 2019–2023 alongside subsidy status in 2021 and 2023. (DOCX)

S1 Text Inclusivity in global research. (DOCX)
