# Supplementary material for: The evolving Lebanese drug crisis: Trends in drug availability and affordability for common outpatient diseases from 2019 to 2023
Source: PLOS Glob Public Health. 2023 Nov 1;3(11):e0002538. doi: 10.1371/journal.pgph.0002538 (PMC10619845; doi:10.1371/journal.pgph.0002538)
Supplement: S1 Table — (DOCX) [file pgph.0002538.s001.docx]

Table Listing Drug Doses, Prices and Availability from 2019-2023 alongside Subsidy Status in 2021 and 2023

D= no longer available on MOPH website, Y= yes, N=no, SH= shortages, RM= raw material subsidized

| **Drug** | **Class** | **Dose** | **8/2019**  **Availab ility** | **8/2019 Price LBP** | **8/2021 Availability** | **8/2021 Price LBP** | **8/2021 Sub-**  **sidy** | **4/2023**  **Availability** | **4/2023 Price LBP** | **4/2023 % Sub-**  **sidy** |
| --- | --- | --- | --- | --- | --- | --- | --- | --- | --- | --- |
| Amoxicillin | Antibiotics | 500mg *10caps | Y | 1372 | Y | 12424 | N | Y | 88290 | 0 |
| Cloxacilline | Antibiotics | 500mg *16 caps | Y | 12868 | N | D | D | N | D | D |
| Amoxicillin + Clavulanic acid | Antibiotics | 1g *14 tab | Y | 11291 | Y | 106186 | N | Y | 652251 | 0 |
| Cefalexin | Antibiotics | 5..mg *12 caps | Y | 11852 | Y | 100525 | N | Y | 714357 | 0 |
| Cefixime | Antibiotics | 400mg *6 caps | Y | 36061 | Y | 326288 | N | Y | 2318676 | 0 |
| Azithromycin | Antibiotics | 250mg *6caps | Y | 11803 | Y | 102848 | N | Y | 640840 | 0 |
| Ciprofloxacin | Antibiotics | 500mg *10caps | Y | 13667 | SH | 120103 | N | Y | 853483 | 0 |
| Doxycycline | Antibiotics | 100mg *15tab | Y | 7843 | SH | 52673 | N | Y | 305856 | 0 |
| Ceftriaxone | Antibiotics | 1G IM inj | Y | 11997 | SH | 10660 | Y | Y | 496651 | 0 |
| Metronidazole | Antibiotics | 500mg *15 tab | Y | 6029 | SH | 47345 | N | Y | 336441 | 0 |
| Nitrofurantoin | Antibiotics | 100mg*20 caps | Y | 7194 | SH | 62611 | N | Y | 424702 | 0 |
| Nnorfloxacin | Antibiotics | 400mg *14tab | Y | 10080 | Y | 43331 | Y | Y | 373685 | 0 |
| Sulfamethoxazole + Trimethoprim | Antibiotics | 160/800mg*20tab | Y | 3962 | Y | 16349 | Y | Y | 124007 | 0 |
| Erythromycin | Antibiotics | 500mg *16 tab | Y | 9632 | N | 47103 | Y | Y | 342891 | 0 |
| Clindamycin | Antibiotics | 300mg *16caps | Y | 11511 | SH | 76438 | N | Y | 518498 | 0 |
| Acenocoumarol | Anticoagulant drugs/ Antithrombotics | 4mg *20tab | Y | 6247 | SH | 11057 | Y | Y | 318756 | 0 |
| Enoxaparin | Anticoagulant drugs/ Antithrombotics | 4000IU/0.4ml *2amp | Y | 22138 | SH | 26066 | Y | Y | 961260 | 0 |
| Phytomenadione | Anticoagulant drugs/ Antithrombotics | 2mg/0.2ml *5 amp | Y | 8764 | N | 10880 | Y | Y | 487912 | 0 |
| Desmopressin | Anticoagulant drugs/ Antithrombotics | 60mcg*30tab | Y | 46226 | N | 47922 | Y | Y | 1750440 | 0 |
| Rivaroxaban* | Anticoagulant drugs/ Antithrombotics | 10mg *10tab | Y | 52654 | N | 54585 | Y | N | 1475638 | 45 |
| Dabigatran* | Anticoagulant drugs/ Antithrombotics | 110mg *60caps | Y | 144064 | N | 130997 | Y | N | 117078 | Y |
| Acetylsalicylic Acid | Anticoagulant drugs/ Antithrombotics | 100mg *20tab | Y | 3152 | Y | 5103 | Y | Y | 158754 | 0 |
| Clopidogrel | Anticoagulant drugs/ Antithrombotics | 75mg *28tab | Y | 56846 | SH | 36928 | Y | Y | 1463737 | 0 |
| Calcium Dobesilate | Anticoagulant drugs/ Antithrombotics | 500mg *30caps | Y | 24942 | Y | 234898 | N | Y | 1434608 | 0 |
| Apixaban* | Anticoagulant drugs/ Antithrombotics | 5mg *60tab | Y | 147668 | N | 142839 | Y | N | 4468003 | 0 |
| Bisoprolol | Cardiovascular drugs | 5mg *30tab | Y | 15471 | SH | 19293 | Y | Y | 269523 | RM 100 |
| Atenolol | Cardiovascular drugs | 100mg *14tab | Y | 11421 | N | 7705 | Y | Y | 302943 | 0 |
| Glyceryl Trinitrate | Cardiovascular drugs | 5mg *10patches | Y | 17243 | N | 12965 | Y | N | 633540 | 0 |
| Isosorbide Dinitrate | Cardiovascular drugs | 40mg *30tab | Y | 8765 | N | 11192 | Y | Y | 251967 | 0 |
| Molsidomine | Cardiovascular drugs | 2mg *30tab | Y | 8963 | SH | 9448 | Y | Y | 431388 | 0 |
| Propranolol | Cardiovascular drugs | 10mg* 50tab | Y | 2259 | Y | 4846 | N | Y | 99871 | 0 |
| Propranolol | Cardiovascular drugs | 40mg *50tab | Y | 3509 | Y | 6790 | N | Y | 124839 | 0 |
| Verapamil | Cardiovascular drugs | 80mg *20tab | Y | 7299 | SH | 9535 | Y | SH | 123184 | RM 100 |
| Digoxin | Cardiovascular drugs | 0.25mg *100tab | Y | 4034 | N | 6319 | Y | SH | 233033 | 0 |
| Amiodarone | Cardiovascular drugs | 200mg *30tab | Y | 11398 | SH | 13640 | Y | Y | 678708 | 0 |
| Amlodipine | Cardiovascular drugs | 5mg *30tab | Y | 13727 | SH | 13090 | Y | Y | 394491 | 0 |
| Diltiazem | Cardiovascular drugs | 60mg *30tab | Y | 4717 | SH | 5985 | Y | Y | 129833 | 0 |
| Ramipril | Cardiovascular drugs | 5mg *30 cpas | Y | 28003 | SH | 21237 | Y | Y | 482711 | 0 |
| Hydrochlorothiazide | Cardiovascular drugs | 25mg * 20tab | Y | 5018 | SH | 9135 | Y | Y | 247181 | 0 |
| Sacubitril / Valsartan | Cardiovascular drugs | 100mg* 28 tab | Y | 134799 | SH | 134799 | Y | N | 128558 | 100 |
| Valsartan | Cardiovascular drugs | 80mg *30tab | Y | 29577 | SH | 19226 | Y | Y | 570930 | 0 |
| Losartan | Cardiovascular drugs | 100mg *30 tab | Y | 38997 | Y | 23806 | Y | Y | 428614 | 0 |
| Methyldopa | Cardiovascular drugs | 250mg *30tab | Y | 7816 | SH | 13898 | Y | Y | 379529 | 0 |
| Captopril | Cardiovascular drugs | 25mg *30tab | Y | 10753 | Y | 19449 | Y | Y | 316259 | 0 |
| Enalapril | Cardiovascular drugs | 10mg *30tab | Y | 8559 | SH | 10666 | Y | Y | 474804 | 0 |
| Furosemide | Cardiovascular drugs | 40mg *20tab | Y | 9330 | SH | 10252 | Y | Y | 131081 | 0 |
| Spironolactone | Cardiovascular drugs | 25mg* 20tab | Y | 4975 | SH | 6918 | Y | Y | 257793 | 0 |
| Fenofibrate | Cardiovascular drugs | 200mg *30 tab | Y | 19113 | SH | 23628 | Y | Y | 345471 | RM 100 |
| Atorvastatin | Cardiovascular drugs | 20mg *30tab | Y | 39493 | Y | 39493 | Y | Y | 953770 | 0 |
| Simvastatin | Cardiovascular drugs | 20mg *30tab | Y | 28800 | Y | 18846 | Y | Y | 224710 | 0 |
| Rosuvastatin | Cardiovascular drugs | 20mg *30tab | Y | 14008 | Y | 18087 | Y | Y | 528485 | 0 |
| Gemfibrozil | Cardiovascular drugs | 600mg *30tab | Y | 10321 | N | 11783 | Y | SH | 987476 | 0 |
| Hydrochlorothiazide , Amiloride HCl | Cardiovascular drugs | 50/5mg *30tab | Y | 9515 | Y | 18024 | N | Y | 470229 | 0 |
| Indapamide | Cardiovascular drugs | 1.5mg *30tab | Y | 10404 | SH | 12576 | Y | Y | 613168 | 0 |
| Gliclazide | Diabetes | 60mg *30tab | Y | 10535 | SH | 9320 | Y | Y | 415090 | 0 |
| Glimepiride | Diabetes | 2mg *30tab | Y | 12003 | Y | 13604 | Y | Y | 249678 | 0 |
| Intermediate-Acting Insulin 70/30 | Diabetes | 100IU/ml *10ml | Y | 11239 | SH | 9636 | Y | SH | 177973 | 60 |
| Long-Acting Insulin N | Diabetes | 100IU/ml *10ml | Y | 11239 | SH | 9636 | Y | SH | 177973 | 60 |
| Rapid-Acting Insulin R | Diabetes | 100IU/ml *10ml | Y | 11239 | SH | 9636 | Y | SH | 177973 | 60 |
| Metformin | Diabetes | 1000mg *30tab | Y | 11041 | SH | 13557 | Y | Y | 193575 | RM 100 |
| Vidagliptin* | Diabetes | 50mg *28tab | Y | 31322 | SH | 33898 | Y | SH | 1460824 | 0 |
| Sitagliptin* | Diabetes | 100mg *28tab | Y | 50224 | SH | 58514 | Y | Y | 3594531 | 0 |
| Dapagliflozin* | Diabetes | 10mg *28tab | Y | 92481 | N | 79486 | Y | N | 2719263 | 0 |
| Canagliflozin* | Diabetes | 100mg *30tab | Y | 89318 | N | 89318 | Y | N | 1758030 | 65 |
| Empagliflozin* | Diabetes | 10mg *30tab | Y | 97659 | SH | 79854 | Y | SH | 5138373 | 0 |
| Insulin LISPRO* | Diabetes | 100IU*ml *5pens | Y | 82995 | N | 80150 | Y | N | 2741978 | 0 |
| Insulin Glulisine* | Diabetes | 100IU/ml *10ml | Y | 39181 | N | 39181 | Y | N | 1404250 | 0 |
| Insulin degludec* | Diabetes | 100IU/ml *5pens 3ml | Y | 154070 | N | 138759 | Y | N | 4139738 | 0 |
| Insulin detemir* | Diabetes | 100IU/ml *5pens 3ml | Y | 116418 | N | 118342 | Y | N | 3156519 | 0 |
| Insulin glargine* | Diabetes | 100IU/ml *10ml | Y | 66864 | N | 66864 | Y | N | 2396391 | 0 |
| Chlorpromazine | Psychiatry | 100mg *50tab | Y | 6700 | Y | 11749 | Y | Y | 344556 | 0 |
| Haloperidol | Psychiatry | 2mg *30tab | Y | 3574 | Y | 6901 | Y | Y | 163955 | 0 |
| Risperidone | Psychiatry | 1mg *20tab | Y | 9806 | SH | 8613 | Y | SH | 332072 | 0 |
| Zuclopenthixol Decanoate | Psychiatry | 200mg/ml amp | Y | 10136 | N | 12706 | Y | Y | 554909 | 0 |
| Clozapine | Psychiatry | 100mg *60tab | Y | 20635 | SH | 27950 | Y | Y | 1606470 | 0 |
| Amitriptyline | Psychiatry | 10mg *100tab | Y | 8381 | Y | 14799 | Y | Y | 409469 | 0 |
| Fluoxetine | Psychiatry | 20mg *30caps | Y | 9462 | SH | 14460 | Y | Y | 728228 | 0 |
| Sertraline | Psychiatry | 50mg *15tab | Y | 11375 | N | 13611 | Y | Y | 646666 | 0 |
| Imipramin | Psychiatry | 10mg *60tab | Y | 7260 | N | 12621 | Y | N | 377014 | 0 |
| Carbamazepine | Psychiatry | 200mg *50tab | Y | 12973 | SH | 12252 | Y | SH | 590412 | 0 |
| Lithium Carbonate | Psychiatry | 400mg *100tab | Y | 23801 | SH | 23801 | Y | Y | 1169533 | 0 |
| Valproic Acid | Psychiatry | 500mg *40tab | Y | 12747 | SH | 15050 | Y | SH | 764639 | 0 |
| Clonazepam | Psychiatry | 0.5mg *50tab | Y | 7945 | SH | 10023 | Y | SH | 438393 | 0 |
| Diazepam | Psychiatry | 5mg *25tab | Y | 4309 | N | 6222 | Y | N | 217012 | 0 |
| Clomipramine | Psychiatry | 10mg *30tab | Y | 5503 | SH | 7831 | Y | N | 322719 | 0 |
| Beclometasone | COPD drugs | 250mcg *200doses | Y | 19877 | SH | 24448 | Y | Y | 1264203 | 0 |
| Budesonide | COPD drugs | 200mcg *200doses | Y | 51223 | N | 51223 | Y | Y | 2142372 | 0 |
| Budesonide + Formoterol | COPD drugs | 160/4.5mcg *60doses | Y | 39362 | N | 32844 | Y | N | 1373656 | 0 |
| Ipratropium Bromide | COPD drugs | 20mcg *200doses | Y | 11005 | SH | 13623 | Y | SH | 464609 | 0 |
| Montelukast | COPD drugs | 10mg *28tab | Y | 23643 | N | 25924 | Y | Y | 1591905 | 0 |
| Salbutamol | COPD drugs | 100mcg *200doses | Y | 5223 | SH | 9515 | Y | Y | 261330 | 0 |
| Aminophylline | COPD drugs | 100mg *100tab | Y | 5751 | SH | 10275 | Y | Y | 289626 | 0 |
| Dextromethorphan | COPD drugs | 27mg/15ml *200ml | Y | 8009 | Y | 67430 | N | Y | 479174 | 0 |
| Tiotropium Bromide* | COPD drugs | 2.5mcg *60doses | Y | 59945 | SH | 62142 | Y | Y | 2598680 | 0 |
| Fluticasone* | COPD drugs | 125mcg *60doses | Y | 16839 | N | 22442 | Y | Y | 1089428 | 0 |
| Fluticasone + Formoterol * | COPD drugs | 125/5mcg *120doses | Y | 77219 | N | 66431 | Y | N | 2380856 | 45 |
| Fluticasone Furoate + Vilanterol* | COPD drugs | 100/25mcg *30doses | Y | 69397 | N | 77178 | Y | N | 2640307 | 45 |
